# Prevalence, correlates, and trajectory of screen viewing among Chinese children in Changsha: a birth cohort study

**DOI:** 10.1186/s12889-022-13268-9

**Published:** 2022-06-11

**Authors:** Chao Li, Gang Cheng, Simin He, Xiaowei Xie, Gang Tian, Ni Jiang, Xianying Min, Yan Shi, Rui Li, Tong Zhou, Yan Yan

**Affiliations:** Department of Epidemiology and Medical Statistics, Xiangya School of Public Health, Central South University, Changsha, 410000 Hunan China

**Keywords:** Screen time, Children, Media use, Cohort study, Outdoor play

## Abstract

**Background:**

High screen viewing time has detrimental effects on children’s health, development, and behavior developing. Children are being exposed to more and more media devices at an earlier age. This study was aimed to determine the amount of daily screen time and its variation and to assess potential factors of screen time by identifying the trajectory of screen time among children aged 1 to 5 years.

**Method:**

This study was based on a representative sample of Changsha young children from a cohort study during 2015–2020. The demographic information and children’s screen viewing time were collected by parents or caregivers through face-to-face interviews. The Latent growth model was used to test the effects of outdoor play on screen viewing time at eight time points, meanwhile, unconditional and conditional models were examined sequentially.

**Result:**

After excluding respondents with missing key variables, we included 953 children in the final analysis. Children’s outdoor play was slightly increased at 18 months and subsequently declined at 24–60 months, with a maximum duration of 2.96 h per day. Children’s average screen time was increased at 18–36 months, and decreased at 42–54 months, with a slight increase at 60 months. The duration of media exposure peaked at 1.4 h/d at age of 36 months and 60 months. Standardized coefficients of the outdoor play at age of 12 months showed negative effects on the screen time in children, but with positive influence at age of 24, 36, and 42 months (*P* <  0.01).

**Conclusion:**

High proportions of young Chinese children in Changsha had more screen time than the AAP recommended according to our analysis. Significant predictors of screen time included pregnancy computer use, paternal educational level, and outdoor play in this study, however, further understanding of risk factors is needed to promote great public health efforts to reduce children’s screen exposure.

**Supplementary Information:**

The online version contains supplementary material available at 10.1186/s12889-022-13268-9.

## Background

Lack of physical activity has been identified as a major cause of preventable morbidity and mortality globally [1–3]. However, unlike inactivity [4, 5], there is less evidence of risk of high levels of sedentary behavior. Sedentary lifestyle is regarded as an independent risk factor for children’s obesity, poorer mental health, and higher blood pressure [6]. However, the evidence for the detrimental impacts of sedentary behavior on children remains fragile. Screen use is a low energy expenditure activity that people do in their leisure time and is therefore considered a form of sedentary behavior [4]. Screen media use can certainly enhance life experiences and leanings in young [7], however, it is important that it is used appropriately. Excessive media use may have detrimental impacts on the health and psychological development of children [8], such as increased BMI [9] and cardiovascular diseases [10], decreased cognitive development [11], emotional symptoms [12], behavioral problems [13], and self-control problems [14]. In addition, these adverse effects of screen exposure in early childhood could be long-term or appear later in life [15]. Therefore, the American Academy of Pediatrics (AAP) guidelines recommended that children under 2 years should be discouraged from digital media use or use media of high-quality programs together with their parents, while for children 2 to 5 years of age, they should use less than 1 h/d of screen time of high-quality programs [16]. However, numerous studies suggested that children now are exposed to high levels of screen exposure at a very early age.

Children are in a digital age, both mobile and traditional media devices have been widely used among young children and occupied a substantial fraction of children’s leisure time [17]. More and more children are being exposed to electronics at an earlier and earlier age worldwide. American children in New York aged 1-year-old had 50.58 min of screen use every day on average, and it turned into 106.87 min when children were at age of 3 years [18]. Meanwhile, data from Canada [19], Australia [20], and other European countries [21] indicated that children aged 2–6 years old had an average screen time of about 1.5–7 h per day. Chinese children have also been exposed to media devices at an early age. Preschool children in Shanghai, China were exposed to 2.8 h/day of screen time, with 78.6% exceeding 1 h/day and 53% exceeding 2 h/day [22]. However, lack of research based on a longitudinal study exploring the distribution of screen time and trajectory changes in young children. Besides, we also lack electronic media exposure for children from inland areas like Changsha.

Children who were exposed to screens in early childhood or grow up with media devices at home are likely to develop excessive screen viewing behavior since it is habit-forming [23]. Reducing screen use in early childhood is important for children’s health and development, but potential predictors influencing screen time in Chinese children are not well known since they may be different from children in other countries [24]. Previous studies have shown that higher screen time was related to maternal depression [25], childcare settings [26], parental screen use [27], and racial minority groups [24, 28]. But there were inconsistent associations between maternal age [24], the presence of siblings [29] and parental educational levels [30], and higher screen time in previous studies. Besides, it is essential for researchers to explore the screen use patterns by using a longitudinal framework since children’s screen time could change over time. Many studies [31, 32] examined the longitudinal trajectories of screen time among children aged 5 years or older, with only two studies [18, 27] exploring longitudinal trajectories of screen time among children under 5. To our knowledge, no other studies have tested the longitudinal trajectories of children’s screen time at the beginning of infancy in the mainland of China. Moreover, a single measurement of early screen time is generally used, whereas repeated measures during the early years may provide a stronger indication of longitudinal screen habits.

Given these gaps in the study, this study aimed to determine the daily screen time use and its variation among Chinses children in Changsha, to be more specific: 1. the distribution of children’s screen time and its change over 4 years; 2. the prevalence of children whose screen use exceeding the suggestion of AAP guideline; 3. the rate of different types of electronics used by children at different months; 4. applying a longitudinal trajectory to test the variation of daily screen time over 4 years follow-up. Besides, we explored potential determinants of screen time among children from 1 to 5 years old.

## Methods

### Data and sample

**Fig. 1:**
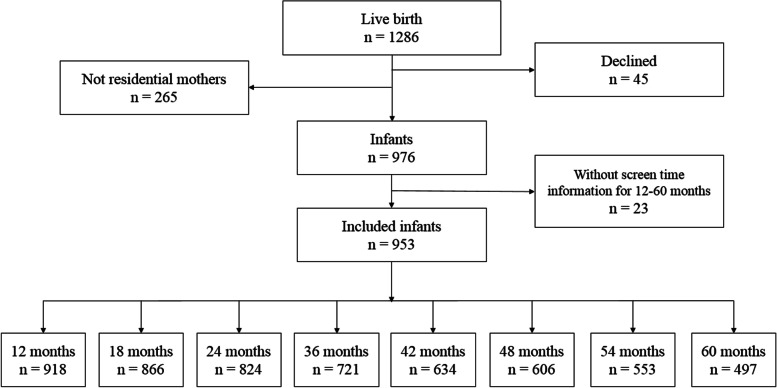
Flow diagram of the participants followed up

This study was based on data derived from a birth cohort study, a population-based survey, which aimed to explore the development of children in their 0–5 years old in Changsha. The study was conducted in three communities in Kaifu District of Changsha from January 2015. Newborn babies in this area with their mother’s Hukou in this area were included, but resident mothers with mental illness or brain diseases or newborns who suffered from severe medical conditions were excluded from this study. A total of 1286 infants were born during 2015, and 976 infants included at the baseline were first conducted through face-to-face interviewing with a respondent rate of 75.9%, and these participants were followed at the age of 3, 6, 8, 12, 18, 24, 36, 42, 48, 54, 60 months. The questionnaires were administered to the caregivers through home visits when children were at the age of 1–54 months, and the caregivers were asked to answers the questionnaires through phone visits and finish online questionnaires when their children were at 54–60 months because of the reduced direct contact due to COVID-19 epidemic. The children’s daily screen time data were collected at 12–60 months, children’s daily outdoor activity time data were collected at 12–60 months, and basic demographic information was at 1–12 months. Children who did not have screen time information for all eight points were excluded. According to the exclusion and inclusion criteria, the cohort recruited 976 participants in the beginning (Fig. 1). Due to the long and frequent follow-ups of the cohort study, there is inevitably a certain amount of missed follow-up. The main reasons for missed follow-up were as follow: 1. the investigators were unable to get in touch with the caregivers due to moving (moving to another city or province), changing contact number; 2. the children’s caregivers were refused to continue to cooperate with the cohort; 3. the children and their caregivers were unable to participate in follow-up visits at a certain point in time due to special family circumstances (for example, a family member (other than the child in the cohort) has an unexpected health problem that requires a companion from their families at some point in time). The cohort study has been performed in accordance with the Declaration of Helsinki. Written informed consent was provided by each participant, and the cohort study was approved by the Independent Ethics Committee Institute of Clinical Pharmacology, Central South University, Changsha, China (Project number: CTXY-130041-3-2).

### Measurement

#### Assessment of children’s daily screen time and outdoor activity time

Children’s daily screen time and outdoor activity time were reported by parents or caregivers in face-to-face interviews. Parents or caregivers were asked to report the “number of hours of screen time on a typical day” at the age of 12–36 months and to report the “number of hours of screen time on every weekday or weekend” at the age of 42–60 months. The data was calculated into daily screen time by using the formula “(weekday screen time *5 + weekend screen time *2)/7”. Since the AAP guidelines suggested that children under 2 years should not be exposed to media devices, children aged 2–5 should use less than 1 h/d of screen time, we divided children’s daily screen time into 2 groups: accepting AAP guidelines and exceeding AAP guidelines according to the reported daily screen use. Meanwhile, parents and caregivers reported the “number of hours of daily outdoor activity time” when the children were at the age of 12–36 months and reported the “number of hours of weekday and weekend outdoor activity time” at the age of 42–60 months. The data was transferred into daily outdoor activity time by calculating through the formula “(outdoor activity time *5 + outdoor activity time *2)/7” for the analysis.

#### Assessment of covariates

According to previous studies [18, 21, 24], potential covariates at baseline included in our analysis were sociodemographic factors, paternal factors, and maternal factors, such as children’s gender, paternal and maternal age, paternal and maternal occupation, and family monthly income. The pregnancy depressive symptoms of children’s mothers were evaluated by using the Edinburgh Postpartum Depression Scale. This scale has been used to evaluate the pregnancy depression when children were in their 3 months. Each item was scored on a 3-point with a total maxim score of 30 [25]. The definitions of variables are summarized in Table 1.

**Table 1:** Definitions of variables used

Baseline characteristics	Wave	Definition
Time-invariant	Wave 1 (children in 12 months)	
Children gender		Male = 1, female = 2
Family member number		Continuous
Pregnant weight (kg)		Continuous
Physical activity in pregnant period (minutes/day)		Continuous
Pregnancy exercise		Yes = 1, no = 2
Pregnancy exercise intensity		Low intensity exercise = 1, moderate exercise = 2
Mother physical activity behavior		Yes = 1, no = 2
Mother physical activity		Low intensity exercise = 1, moderate exercise = 2, heavy exercise = 3
Pregnancy computer use		Yes = 1, no = 2
Pregnancy phone use		Yes = 1, no = 2
Maternal race (Han)		Ethnic Han = 1, not Ethnic Han = 2
Maternal age		Continuous
Educational level		Less than primary school = 1, middle school = 2, high school = 3, university = 4, graduate school = 5, other education = 6
Maternal occupation		Complete collection of Chinese Occupational Classification: class 1 = 1, class 2 = 2, class 3 = 3, class 4 = 4, class 5 = 5, class 6 = 6
Paternal race (Han)		Ethnic Han = 1, not Ethnic Han = 2
Paternal age		Continuous
Educational level		Less than primary school = 1, middle school = 2, high school = 3, university = 4, graduate school = 5, other education = 6
Paternal occupation		Complete collection of Chinese Occupational Classification: class 1 = 1, class 2 = 2, class 3 = 3, class 4 = 4, class 5 = 5, class 6 = 6
Infant weight (kg)		Continuous
Household income (yuan/month)		Less than 2000 yuan/month = 1, 2001–5000 yuan/month = 2, 5001–10,000 yuan/month = 3, 10,001–15,000 yuan/month = 4, More than 15,000 yuan/month = 5
Pregnancy depression		Not depression = 0, depression = 1
Time-variant	Wave 1–8 (from children in 12 months to 60 months)	
Children’s daily screen time		Continuous
Children’s daily screen time prevalence		Accept AAP guideline (the reported amount of screen use among children was no more than the recommended screen time of AAP guidelines) = 0, exceed AAP guideline (the reported amount of screen use among children was more than the recommended screen time of AAP guidelines) = 1
Prevalence of children’s interest in screen use		No = 1, occasionally = 2, sometimes = 3, often = 4, always = 5
Children’s daily outdoor activity time		Continuous

### Data analysis

#### Descriptive statistics

Mean (Standard Deviation) or number and percentage were reported to summarize children’s outdoor play and screen-time, and other continuous factors. Children’s screen time was analyzed as both continuous and binary outcome variables. When compared with the recommendations of daily screen time of AAP guidelines, children were divided into two groups: 1. accepting AAP guidelines group, which means that children under 2 years had no screen time or children at age of 2–5 years used media devices for less than 1 h per day; 2. exceeding AAP guidelines group, which means that children under 2 years had screen use or children aged 2–5 years used more than 1 h of media devices per day. Spearman’s coefficients were used to explore the correlations between children’s daily outdoor activity time and daily screen time at eight waves. One-way repeated-measures analysis of variance (ANOVA) was used to assess the changes of screen use patterns, including duration and frequency of screen time, rate of children’s interest in screen time, and duration of outdoor play in eight waves.

#### Latent growth model

LGMs were used to examine the trajectories of changes in children’s screen time and children’s outdoor play. The trajectory of change in screen use across time was modeled with two latent variables: latent intercept growth factor, representing the initial status of screen-time, and latent slope growth factor, reflecting the rate of change in screen time. A specified linear LGM was modeled by the growth trajectory of the variable with our data.

**Fig. 2:**
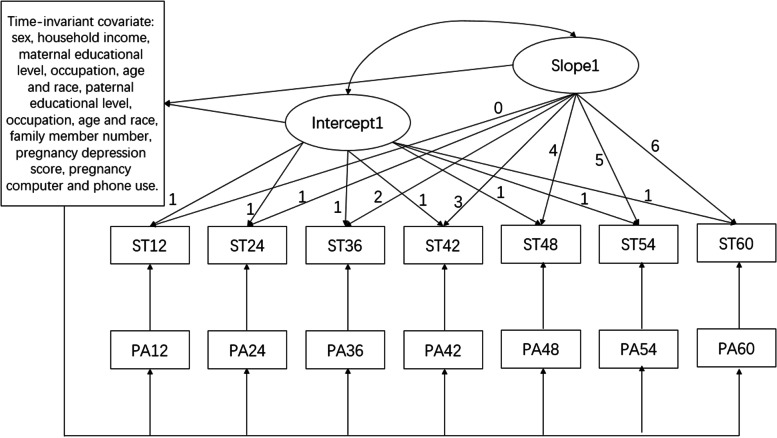
Measurement of latent growth model with time-invariant and time-variant covariates for screen time. (Time-invariant covariates include age, family member number, family income, pregnancy computer use, pregnancy phone use, pregnancy depression symptom, maternal age, maternal educational level, maternal occupation, maternal race, paternal age, paternal educational level, paternal occupation, paternal race. PA Outdoor play, ST Screen time)

We established an initial unconditional LGM model to reflect the change of children’s daily screen-time over time with a time-variant variable of children’s outdoor playtime at eight-time points. The loading from the intercept factor in the initial model to each of the repeated measures is a fixed value of 1.0. For the slope factor, we fixed the loading of the children’s screen time at the values of 0, 1.0, 2.0, 3.0, 4.0, 5.0, 6.0, 7.0, and 8.0. The initial model was compared with the conditional model, which was based on the initial model with adjustments of a series of predictor variables (Fig. 2).

**Fig. 3:**
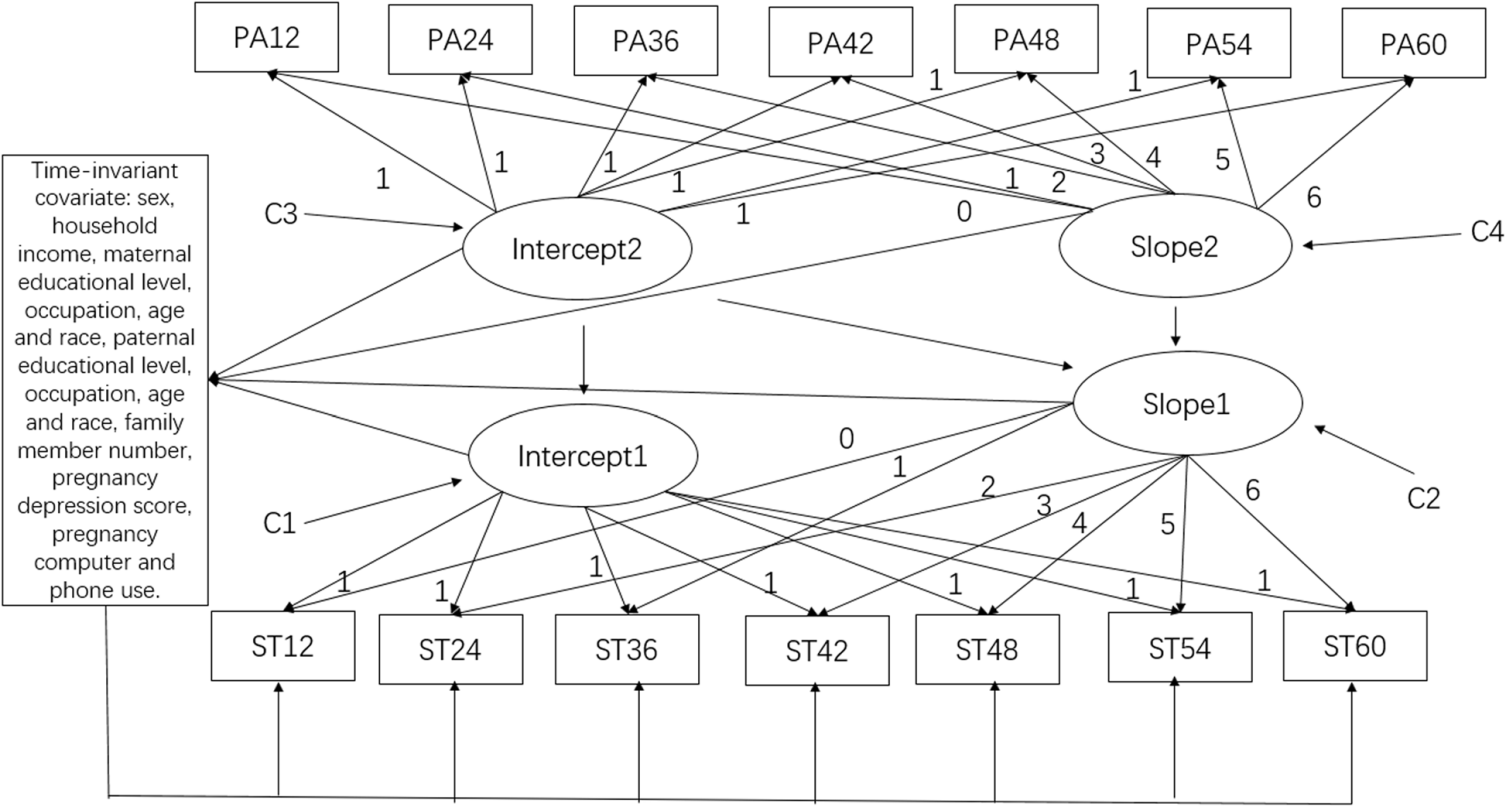
Conditional structural latent growth model to assess the relationships of the changes in outdoor play (PA) and screen time (ST). (C1-C2 Correlation of the intercept 1 and slope 1, C3-C4. Correlation of the intercept 2 and slope 2)

Then we assess the relationship of the changes in outdoor play with changes in screen time. Two unconditional LGMs were applied to present the changes in the trajectories of screen time and outdoor play, separately, with no adjusted covariates. The intercept and slope factors values were set similarly to the initial model. Unconditional and conditional (including predictors) models were compared sequentially to test the impacts of outdoor play on the initial level and subsequent development of screen time. Age, family member number, family income, pregnancy computer use, pregnancy phone use, pregnancy depression symptom, maternal age, maternal educational level, maternal occupation, maternal race, paternal age, paternal educational level, paternal occupation, paternal race were served as predictors in the conditional model (Fig. 3).

We used the following indices to assess the goodness of model fit: chi-square statistic, standardized root mean square residual (SRMR) ≤ 0.50, root mean square error of approximation (RMSEA) ≤ 0.08, comparative fit index (CFI) ≥ 0.95, and Tucker-Lewis index (TLI) ≥ 0.95 [33]. We assumed that data were missing at random in this analysis. The LGMs were conducted with a maximum likelihood estimator [34–36]. By building LGMs, missing data was not imputed, but participants with missing data at some time points were included in the models. The statistical analyses were conducted by SPSS 22.0 and Mplus 8.3.

## Results

### Descriptive analysis

After respondents without screen time data were derived from the cohort sample, the present study included 953 participants, in the final analysis, 24 respondents without any screen use data were excluded. Compared with the excluded sample, included sample’s mothers were more likely to have outdoor activity behavior (58.3% VS 29.8%, *P* = 0.032). There were no other significant differences in respondents’ baseline characteristics between the included sample and excluded participants (Table 2). Table 3 shows the correlations of children’s outdoor play time with screen time when children at 12 months to 60 months. It presents that the duration of outdoor play was negatively associated with screen use at many different time points. To examine this negative correlation between children’s outdoor play and screen time in a cohort study, further analyses were conducted.

**Table 2:** Parental, child, and household characteristics of sample

Sociodemographic Characteristic	Analytic Group		Excluded Group		*P* value
	Observed(*n* = 953)		Observed(*n* = 23)		
	Mean ± SD / %	N	Mean ± SD / %	N	
Children gender					0.642
Male	492 (51.6)	953	13 (56.5)	23	
Female	461 (48.4)		10 (43.5)		
Family member number	4.31 (0.95)	953	4.0 (0.8)	23	0.166
Pregnant weight (kg)	68.38 (9.11)	953	69.6 (10.4)	23	0.525
Physical activity in pregnant period (minutes/day)	46.69 (25.94)	953	47.2 (22.2)	23	0.93
Pregnancy exercise (yes)	820 (86.9)	944	18 (81.8)	22	0.49
Pregnancy exercise intensity					0.791
Low intensity exercise	810 (99.6)	813	19 (100)	19	
Moderate exercise	3 (0.4)		0 (0)		
Mother physical activity behavior	275 (29.8)	923	7 (58.3)	12	**0.032**
Mother physical activity		278		7	0.651
Low intensity exercise	224 (80.6)		5 (71.4)		
Moderate exercise	46 (16.5)		2 (28.6)		
Heavy exercise	8 (2.9)		0 (0)		
Pregnancy computer use	318 (80.7)	383	0 (0)	5	0.551
Pregnancy phone use	323 (96.7)	334	4 (100)	4	0.712
Maternal race (Han)	921 (96.6)	944	23 (100)	23	0.671
Maternal age (years)	29.92 (3.92)	953	30.6 (4.7)	23	0.398
Educational level		942		22	0.721
Less than primary school	2 (0.2)		0 (0)		
Middle school	31 (3.3)		0 (0)		
High school	114 (12.1)		4 (18.2)		
University	722 (76.6)		15 (68.2)		
Graduate school	69 (7.3)		3 (13.6)		
Other education	4 (0.4)		0 (0)		
Maternal occupation		940		22	0.787
1	35 (3.7)		0 (0)		
2	178 (18.7)		6 (26.1)		
3	365 (38.3)		8 (34.8)		
4	2 (0.2)		0 (0)		
5	5 (0.5)		0 (0)		
6	355 (37.3)		8 (34.8)		
Paternal race (Han)	920 (96.5)	943	23 (100)	23	0.364
Paternal age (years)	32.06 (4.85)	953	33.0 (6.9)	23	0.384
Educational level		941		23	0.725
Less than primary school	4 (0.4)		0 (0)		
Middle school	25 (2.7)		1 (4.3)		
High school	109 (11.6)		2 (8.7)		
University	690 (73.3)		15 (65.2)		
Graduate school	108 (11.5)		5 (21.7)		
Other education	5 (0.5)		0 (0)		
Paternal occupation		933		23	0.658
1	55 (5.8)		1 (4.3)		
2	240 (25.2)		3 (13)		
3	303 (31.8)		7 (30.4)		
4	3 (0.3)		0 (0)		
5	42 (4.4)		2 (8.7)		
6	290 (30.4)		10 (43.5)		
Infant weight (kg)	3.33 (0.46)	953	3.37 (0.57)	23	0.686
Household income (yuan/month)					
Less than 2000 yuan/month	31 (3.4)	922	0 (0)	23	0.259
2001–5000 yuan/month	493 (53.5)		9 (39.1)		
5001–10,000 yuan/month	360 (39.0)		14 (60.9)		
10,001–15,000 yuan/month	28 (3.0)		0 (0)		
More than 15,000 yuan/month	10 (1.1)		0 (0)		
Pregnancy depression	29 (3.0)	953	0 (0)	23	0.396

**Table 3:** Correlations of screen time with outdoor play in children at eight time point

	ST12	ST18	ST24	ST36	ST42	ST48	ST54	ST60	PA12	PA18	PA24	PA36	PA42	PA48	PA54	PA60
ST12	1															
ST18	0.269^**^	1														
ST24	0.157^**^	0.377^**^	1													
ST36	0.132^**^	0.163^**^	0.315^**^	1												
ST42	0.203^**^	0.267^**^	0.246^**^	0.427^**^	1											
ST48	0.188^**^	0.191^**^	0.264^**^	0.308^**^	0.565^**^	1										
ST54	0.134^**^	0.187^**^	0.247^**^	0.291^**^	0.434^**^	0.519^**^	1									
ST60	−0.055	0.076	0.068	0.087	−0.008	0.018	0.07	1								
PA12	0.027	0.019	−0.029	0.002	0.066	0.103*	0.076	0.026	1							
PA18	0.067	−0.074	−0.081^**^	−0.068	−0.06	−0.049	0.039	−0.001	0.307^**^	1						
PA24	0.067	0	−0.03	0.019	−0.007	0.015	−0.04	−0.032	0.226^**^	0.264^**^	1					
PA36	0.01	−0.014	−0.008	0.052	−0.05	− 0.071	− 0.003	0.087	0.225^**^	0.201^**^	0.27^**^	1				
PA42	0.052	−0.013	0.038	−0.069	0.099^*^	0.11*	0.004	−0.01	0.088^*^	0.119^**^	0.104^*^	0.091^*^	1			
PA48	−0.04	− 0.102^*^	− 0.088^*^	− 0.127^**^	− 0.062	0.026	− 0.05	−0.009	0.057	0.114^**^	0.097^*^	0.044	0.227^**^	1		
PA54	−0.116^**^	− 0.066	−0.012	− 0.006	−0.016	− 0.047	0.055	0.154^*^	0.013	−0.051	−0.055	0.098^*^	0.011	0.207^**^	1	
PA60	0.043	0.124^*^	0.002	0.016	0.002	0.063	0.03	0.085	0.02	−0.005	−0.059	0.026	−0.01	− 0.103	0.079	1

*Abbreviations*: *PA* Outdoor activity time, *ST* Screen time

^**^P <  0.01

^*^P <  0.05

Changes in children’s outdoor play, screen time, and media use interests for participants with complete data at each time point at eight-time points are summarized in Table 4. One-way repeated-measures analysis shows significant changes in children’s outdoor play and screen use. During the study period, non-linear changes were observed in both children’s outdoor play and screen time, children’s outdoor play was slightly increased at 18 months and subsequently declined at 24–60 months, with a maximum duration of 2.96 h per day. Children’s average screen time was increased at 18–36 months, and decreased at 42–54 months, with a slight increase at 60 months. The duration of media exposure peaked at 1.4 h/d at age of 36 months and 60 months. The prevalence of children’s interest in media devices was decreased at eight-time points. The prevalence of children exceeding AAP recommendation was increased after children at age of 12 months, and decreased at age of 36 months, presenting a non-linear change at eight points. The minimum prevalence of children exceeding AAP recommendation was 16.3% at 54 months, and the maximum was 84.8% at age of 18 months. There are some descriptions of other screen use patterns in Table 5. The maximum rate of cohort children watching TV was 90.2% at their 42 months while 57.2% of included children using a phone at age of 24 months. Meanwhile, the prevalence of children’s limited media use by parents was higher and higher from 36 months to 60 months (Table 5).

**Table 4:** Levels of screen time and outdoor play in children at each time point

Variable	Time point							
	12 months	18 months	24 months	36 months	42 months	48 months	54 months	60 months
Prevalence of children’s interest in screen use	F = 11,164.509	*P* < 0.001^a^						
Included population number	917	866	824	717	634	606	553	497
NO	139 (15.2%)	123 (14.2%)	48 (5.8%)	16 (2.2%)	7 (1.1%)	5 (0.5%)	7 (1.3%)	12 (2.4%)
Occasionally	205 (22.4%)	263 (30.4%)	268 (32.5%)	81 (11.3%)	81 (12.8%)	74 (12.2%)	69 (12.5%)	85 (17.1%)
Sometimes				133 (18.5%)	161 (25.4%)	149 (37.6%)	110 (13.7%)	117 (23.5%)
Often	573 (62.4%)	480 (55.45%)	508 (61.7%)	292 (40.7%)	283 (44.6%)	248 (40.9%)	225 (40.7%)	217 (43.7%)
Always				195 (27.2%)	102 (16.1%)	130 (21.5%)	142 (25.7%)	66 (13.3%)
Prevalence of children’s acceptance of AAP	F = 278.488	*P* < 0.001^a^						
Included population number	879	692	729	695	561	466	504	466
Not using media device	530 (60.3%)	105 (15.2%)	20 (2.7%)	8 (1.2%)	12 (2.1%)	8 (1.7%)	12 (2.4%)	17 (3.6%)
Accepting AAP recommendation	530 (60.3%)	105 (15.2%)	580 (79.6%)	506 (72.8%)	450 (80.2%)	380 (81.5%)	422 (83.7%)	371 (79.6%)
Exceeding AAP recommendation	349 (39.7%)	587 (84.8%)	149 (20.4%)	189 (27.2%)	111 (19.8%)	86 (18.5%)	82 (16.3%)	95 (20.4%)
Children’s daily screen time	F = 481.461	*P* < 0.001^a^						
Included population number	879	692	729	695	561	466	504	466
Children’s screen time (hours/day)	0.23 (0.66)	0.61 (0.91)	1.02 (0.94)	1.40 (2.14)	1.27 (1.22)	1.27 (1.55)	1.13 (1.00)	1.40 (2.00)
Children’s daily outdoor activity time	F = 5505.670	*P* < 0.001^a^						
Included population number	918	835	787	721	624	598	553	486
Children’s outdoor activity time (hours/day)	2.58 (1.17)	2.96 (1.17)	2.73 (1.23)	2.58 (1.25)	2.02 (0.85)	1.93 (0.69)	1.90 (0.75)	1.87 (0.94)

^a^*P* value was calculated by one-way repeated measures ANOVA

**Table 5:** Screen use patterns in Chinese children in Changsha at each time point

Variable	Time point							
	18 months	24 months	36 months	42 months	48 months	54 months	60 months	*P* value^a^
Included population number	866	824	717	634	606	553	497	
Media device								
Phone	414 (47.8%)	471 (57.2%)	368 (51.3%)	289 (45.6%)	284 (46.9%)	255 (46.1%)	242 (48.7%)	< 0.001
TV	585 (67.6%)	675 (81.9%)	646 (90.1%)	572 (90.2%)	524 (86.5%)	454 (82.1%)	350 (70.4%)	< 0.001
iPad	49 (5.7%)	101 (12.3%)	99 (13.8%)	107 (16.9%)	137 (22.6%)	165 (29.85)	219 (44.1%)	> 0.05
Video game	0	2 (0.2%)	5 (0.7%)	1 (0.2%)	3 (0.5%)	2 (0.4%)	5 (1.0%)	> 0.05
Computer	13 (1.5%)	15 (1.8%)	17 (2.4%)	14 (2.2%)	19 (3.1%)	9 (1.65%)	22 (4.4%)	> 0.05
other	8 (0.9%)	18 (2.2%)	4 (0.6%)	10 (1.6%)	9 (1.5%)	24 (4.3%)	29 (5.8%)	> 0.05
Usage Scenarios								
Education	114 (13.2%)	151 (18.35%)	132 (18.4%)	135 (21.3%)	112 (18.5%)	112 (20.3%)	160 (32.2%)	0.105
Soothing when crying	73 (8.4%)	89 (10.85%)	37 (5.2%)	22 (3.55%)	8 (1.3%)	20 (3.6%)	19 (3.8%)	> 0.05
Substitute caretaker	99 (11.4%)	257 (31.2%)	143 (19.9%)	91 (14.4%)	75 (12.4%)	69 (12.5%)	36 (7.2%)	> 0.05
Children watching while adults use it	349 (40.3%)	229 (27.8%)	109 (15.2%)	50 (7.9%)	58 (9.6%)	62 (11.2%)	82 (16.5%)	> 0.05
other	128 (14.8%)	248 (30.15%)	33 (4.6%)	32 (5.0%)	57 (9.4%)	72 (13.0%)	72 (14.5%)	> 0.05
Limitation on screen time			626 (87.3%)	554 (87.4%)	558 (92.1%)	516 (93.3%)	475 (95.6%)	< 0.001

Since children could have more than one media device to use every day, the number of cases does not always sum to the included population number

Since children could have more than one usage scenario every day, the number of cases does not always sum to the included population number

^a^*P* value was calculated by one-way repeated measures ANOVA

### Latent growth model

Table 6 presents the results of the initial LGM and the adjusted LGM. Based on the initial model, the trajectory of the screen time and controlled outdoor activity was described by the specified linear model. The intercept of the screen time was 2.206 h/day (*P* <  0.01), and the slope was 1.449 h/day (*P* = 0.001). Standardized coefficients of the outdoor activity at age of 12 months showed negative effects on the screen time in children, and with positive influence at age of 24, 36, and 42 months(*P* <  0.01). However, these effects were changed in the adjusted model, except for the significant associations presented in the initial model, outdoor activity for children at age of 48 months showed a positive association with screen time(*P* = 0.009). The results of the measurement and structural models are summarized in Supplemental Table 1. The trajectory of the children’s outdoor activity time was depicted by the linear LGM, with good fit indices (Supplemental Table 1). The intercept of the children’s outdoor playtime growth trajectory showing the initial outdoor play time was 4.002 h/day (*P* <  0.001). The estimate of the slope was -1.383h/day (*P* <  0.001), indicating a typical decrease in the average rate of changes in outdoor play across eight waves. The trajectory of screen time was well described with good fit indices. The slope was 3.555h/day (*P* > 0.05), showing a nonsignificant decline in the average rate of change in screen time during the period of this cohort.

**Table 6:** Standardized coefficients for initial model and adjusted latent growth models

Models	Parameters	Standardized coefficients	Standard error	*P* value	Goodness-of-fit indices
Initial model	Intercept	2.206	0.466	**< 0.001**	χ2(7) = 145.588, *P* < 0.001, CFI = 0.679, TLI = 0.584, SRMR = 0.066; RMSEA = 0.033 (0.025, 0.041)
	Slope	1.449	0.431	**0.001**	
	PA12	−0.200	0.05	**< 0.001**	
	PA18	−0.024	0.027	0.379	
	PA24	0.103	0.025	**< 0.001**	
	PA36	0.105	0.019	**< 0.001**	
	PA42	0.102	0.025	**< 0.001**	
	PA48	0.042	0.022	0.05	
	PA54	−0.020	0.038	0.6	
	PA60	0.012	0.029	0.667	
Adjusted models^a^	Intercept	4.405	1.688	**0.009**	χ2(7) = 256.470, *P* < 0.001, CFI = 0.679, TLI = 0.617, SRMR = 0.039; RMSEA = 0.023 (0.017, 0.029)
	Slope	3.848	1.608	**0.017**	
	PA12	−0.120	0.029	**< 0.001**	
	PA18	0.013	0.020	0.505	
	PA24	0.137	0.020	**< 0.001**	
	PA36	0.118	0.018	**< 0.001**	
	PA42	0.123	0.022	**< 0.001**	
	PA48	0.051	0.019	**0.009**	
	PA54	−0.001	0.030	0.967	
	PA60	0.014	0.025	0.579	

*Abbreviations*: *CFI* Comparative fit index, *PA* Outdoor activity time, *RMSEA* Root mean square error of approximation, *SRMR* Standardized root mean square residual, *ST* Screen time, *TLI* Tucker-Lewis index

^a^Adjusted for age, family member number, family income, pregnancy computer use, pregnancy phone use, pregnancy depression symptom, maternal age, maternal educational level, maternal occupation, maternal race, paternal age, paternal educational level, paternal occupation, paternal race

The unconditional structural models were used to assess the relationships of the initial status and changes in children’s outdoor play with initial status and changes in screen time, which were established with the satisfactory model fitness. Based on the results of the unconditional model, it was observed that the initial level of the outdoor play was positively associated with the initial level of screen time at baseline. The path standardized coefficient between two intercepts was nonsignificant (β = 0.224, *P* > 0.05), and the path standardized coefficient of the intercept of outdoor play and slope of screen time was also not significant (β = − 0.21, P > 0.05). Based on the path standardized coefficient of two slope growth factors, the rate of change in outdoor play showed a nonsignificant association with the rate of change in cognitive function (β = 0.187, P > 0.05).

After controlling the predictors, the conditional LGM presented better fit indices than the unconditional model. Consistent with the results of the unconditioned model, there were no significant associations found in this adjusted model. Table 7 shows the standardized coefficients for time-invariant covariates in the conditional LGM. According to the results in Table 7, it can be found that participants with more screen time at initial whose mother had more pregnancy computer use. Children with higher growth rates of screen time had fathers with lower educational levels. Supplemental Table 2 presents the standardized coefficients for covariates in the conditional structural LGM. Based on the results in Supplemental Table 2, it can be easily observed that participants with more outdoor play time were prone to be with younger and Han-race fathers at baseline. In addition, the covariates of paternal age and paternal educational level were identified as the relative factors of changes in outdoor play time.

**Table 7:** Standardized coefficients for covariates in the adjusted latent growth models^a^

Covariates	Screen time	
	Initial status	Slope
Sex	−0.003	−0.018
Family member number	0.016	0.007
Household income^b^	0.000	0.000
Pregnancy depression score	0.095	−0.041
Pregnancy computer use	−0.146^*^	0.034
Pregnancy phone use	−0.044	0.033
Maternal age	−0.001	0.004
Maternal race	−0.066	−0.005
Maternal educational level	0.042	−0.027^*^
Maternal occupation	−0.003	0.002
Paternal age	−0.008	−0.003
Paternal race	−0.115	−0.021
Paternal educational level	−0.021	−0.029*
Paternal occupation	−0.024	−0.003

^a^The table showed the standardized coefficients for covariates adjusted in the adjusted latent growth models in Table 6

^b^Household income, average income per household member per month

^*^Statistical significance

## Discussion

The results were based on data from a birth cohort that was a representative sample of Chinese children in Changsha. For Chinese children in Changsha, their daily duration of screen use increased with the advancement of age, but their daily duration of outdoor play decreased as children grew up. Longer time spent on outdoor activities was associated with less screen use when children at 12 months, but less time spent on outdoor activities was related to less screen time for children at the age of 24 months to 48 months.

Our study found that the duration of screen use had an upward trend from 12 months to 60 months. Meanwhile, our study presented that children had poor adherence to the AAP guidelines, which was consistent with previous work. A study conducted in 2005 observed that 70% 0–2 years old included children did not accept the AAP guidelines [37]. As the children have grown up, the daily duration of media use for children has been increased, however, the prevalence of children who were not complying with media use recommendations of AAP guidelines had not always increased at different time points. The rate of children who exceeded the screen use recommendations of AAP guidelines increased when children were at age of 1 to 2 years old but declined from 3 to 5 years, since AAP guidelines suggest screen time differently between children under 2 years and children aged 2–5 years old. Another study indicated similar results that media use increased until preschool age and declined from 3 to 7 years old [38]. To be more specific, the results of our analysis that the prevalence of included children excepted the AAP guidelines at 18 months of age was the highest, declined in the following months, and the rate reached its lowest point at 54 months of age. This is probably because there were more limitations for children’s screen use by their caregivers from the information collected by the face-to-face follow-up interviews from their 36 months to 60 months. Meanwhile, the bias could be caused by the missing data during these time points, since the caregivers who refused to continue the follow-up or missed follow-ups at some time points might pay less attention to or set fewer limitations on their children’s unhealthy behavior including long screen time and short outdoor play time. The prevalence of children’s adherence to the AAP guidelines in their 54–60 months increased due to our results, and this is probably because of increased screen time during the isolation period at home caused by the COVID-19 panic. COVID-19 panic has been spread around since 2020, and the Chinese were isolated at home at that period [39–41]. Media use became the main activity for Chinese during the isolation period at home since they had much more leisure time to consume, with their children forbidden for outdoor play [39]. In addition, media devices might be used as digital babysitters since their parents had to work from home during isolation periods. Besides, there might be an underestimation of children’s screen use at their 54–60 months due to the large missing data.

The media devices that children used were also an important screen use pattern. We explored the children’s use of mobile and traditional media devices, especially two main kinds, TV and phone. The results presented that children used TV more than phone during the eight time-point follow-ups. TV was regarded as the main media device that children could get attached to according to the previous studies [29, 42, 43], for children in both developed countries [29, 42] and developing countries [43, 44]. Our findings suggested that TV is the main media device among children in Changsha, China. To be more specific, a maximum of 90.2% of children under 5 watched TV during their reported screen time when they were at their age of 42 months, and the rate of children watching TV reached a minimum of 67.6% when they were 12 months. These findings could help us to suggest that children should still be focused on their TV viewing even the portable media devices are getting popular in early childhood. As for the mobile media devices, a fluctuation curve was found during the period. A maximum prevalence of 57.2% of children used phones at age of 24 months, and 45.6% of children at age of 42 months used phones during their reported screen time at a minimum rate. These are novel descriptions of phone viewing time for children from 12 months to 60 months since no previous studies have described it in Chinese children.

Several covariates were tested and adjusted, specifically the baseline potential factors and a time-variant variable. Pregnancy computer use was negatively associated with children’s average screen time in the beginning, and Paternal educational level had a negative association with the growth rate of screen time during the follow-up period. Pregnancy computer use may be reflective of mothers’ attitudes on screen use, it could have effects on children’s screen use when they were 12 months, but its influence might weaken as children grow up. With inconsistent associations found by previous studies, the results from this analysis could enhance the evidence on negative associations between paternal education and higher screen use on long-term influence. Children whose fathers with lower educational levels were more likely to have faster growth rates of screen viewing time. Outdoor play was regarded as an essential time-variant covariate in our analysis according to previous analyses [45, 46]. We adjusted other covariates to figure out how this time-invariant covariate could affect children’s screen viewing time. From our results, a negative association between outdoor activity and screen time was found at age of 12 months, however, a positive association was found at age of 24–48 months. When children are at the age of 12 months, sleep an average of 12–13 h at night, and the night sleep duration decreases with the advancement of age [47]. The screen time would decrease if their outdoor play increased since the total wake-up time for toddlers was short and they do not have many kinds of activities to spend their leisure time [48]. In addition, caregivers are also becoming aware of the importance of outdoor activities and the limitations of electronic screens, as the children get older [49]. It is unclear why more outdoor play would be related to more screen time in children during their 24 months to 48 months.

The prevalence of children’s interests in screen use was also non-linear. 62.4% of children at 12 months were interested in media devices, which was the highest rate. The cause of this situation is that children are very curious about the world and electronic screens at this age, with the brilliant pictures and sounds of media devices attracting their attention [16, 50]. The rate of children’s interests in media devices declined in the following months and reached 40.7% as a minimum at the age of 36 months. The majority of included children entered kindergarten at the age of 36 months, friends at the same age, and new knowledge would distract the children’s interests from media use. In this case, parental accompaniment with some family games and outdoor activities would be very important for children [51], especially for younger children to keep children away from electronics. Except that, the large amount of missing data at later time points could cause the loss of data on children who exceed the AAP guidelines, which could lead to an underestimation of the results, especially at later time points.

Despite the strengths, including the long-term follow-ups, repeated reported screen use information which was collected through face-to-face interviews with sociodemographic and behavioral predictors, this analysis had some limitations. Screen time data were based on parents’ or caregivers’ reports rather than 24-h recall diary or direct observation. This could cause some inaccuracies since the background screen viewing time or screen use in daycare or kindergarten might be overlooked by their parents or caregivers. However, both qualitative and objective screen time measurements are challenging and costly assessments in large longitudinal studies. Besides, the validity and reliability of the questionnaire used in our study are unknown. Finally, our cohort in Changsha is not representative of the entire Chinese children. Therefore, the screen time pictures observed in our study are different from those at a national level.

## Conclusion

In summary, high proportions of children of all ages were exposed to screen use which exceeded AAP guidelines. We observed that TV viewing was still the main part of screen time and the prevalence of using phones among children did not have a big change at all ages. Significant predictors of screen time included pregnancy computer use, paternal educational level, and outdoor play, which could be used for health promotion activities and screen time interventions for children in Changsha. Future interventions should focus on Chinese children in Changsha under 36 months since the rate of children who had screen use exceeding AAP guidelines under 36 months was high. Further understanding of risk factors is needed to promote great public health efforts to reduce children’s screen exposure in China.

## Supplementary Information

**Additional file 1: Supplemental Table 1.** Standardized coefficients for measurement and structural models. **Supplemental Table 2.** Standardized coefficients for covariates in the adjusted structural latent growth model.

## Data Availability

All data generated or analyzed during this study are included in this article.
