# Supplementary material for: Prevalence, correlates, and trajectory of screen viewing among Chinese children in Changsha: a birth cohort study
Source: BMC Public Health. 2022 Jun 11;22:1170. doi: 10.1186/s12889-022-13268-9 (PMC9188020; doi:10.1186/s12889-022-13268-9)
Supplement: Supplementary file 1 — Additional file 1: Supplemental Table 1. Standardized coefficients for measurement and structural models. Supplemental Table 2. Standardized coefficients for covariates in the adjusted structural latent growth model. [file 12889_2022_13268_MOESM1_ESM.docx]

Supplemental file

Supplemental Table 1. Standardized coefficients for measurement and structural models

Supplemental Table 2. Standardized coefficients for covariates in the adjusted structural latent growth model

Supplemental Table 1. Standardized coefficients for measurement and structural models

| Models | Parameters | Coefficients | P value | Goodness-of-fit indices |
| --- | --- | --- | --- | --- |
| Measurement models |  |  |  |  |
| Trajectory of screen time | Intercept | 2.567 | 0.043 | χ2(7) = 315.581, P < 0.001, CFI = 0, TLI = -0.249, SRMR = 0.137; RMSEA = 0.098 (0.088, 0.108) |
|  | Slope | 3.555 | 0.066 |  |
| Trajectory of PA | Intercept | 4.002 | < 0.001 | χ2(7) = 326.138, P < 0.001, CFI = 0.131, TLI = 0.215, SRMR = 0.090; RMSEA = 0.100 (0.090, 0.110) |
|  | Slope | -1.383 | < 0.001 |  |
| Structural models |  |  |  |  |
| Unconditional model | PA intercept → ST intercept | 0.224 | 0.866 | χ2(7) = 731.722, P < 0.001, CFI = 0, TLI = -0.049, SRMR = 0.094; RMSEA = 0.072 (0.067, 0.078) |
|  | PA intercept → ST slope | -0.210 | 0.872 |  |
|  | PA slope → ST slope | 0.187 | 0.895 |  |
|  |  |  |  |  |
| Conditional model | PA intercept → ST intercept | 0.021 | 0.933 | χ2(7) = 1080.949, P < 0.001, CFI = 0, TLI = -0.282, SRMR = 0.063; RMSEA = 0.050 (0.047, 0.053) |
|  | PA intercept → ST slope | 0.337 | 0.265 |  |
|  | PA slope → ST slope | -1.508 | 0.280 |  |

Abbreviations: CFI Comparative fit index, PA Outdoor physical activity time, RMSEA Root mean square error of approximation, SRMR Standardized root mean square residual, ST Screen time, TLI Tucker-Lewis index

^a^Adjusted for age, family member number, family income, pregnancy computer use, pregnancy phone use, pregnancy depression symptom, maternal age, maternal educational level, maternal occupation, maternal race, paternal age, paternal educational level, paternal occupation, paternal race

Supplemental Table 2. Standardized coefficients for covariates in the adjusted structural latent growth model

| Covariates | Screen time | | Outdoor play time | |
| --- | --- | --- | --- | --- |
|  | Initial status | Slope | Initial status | Slope |
| Sex | -0.007 | -0.175 | -0.082 | 0.015 |
| Family member number | 0.091 | 0.151 | 0.030 | -0.009 |
| Household income | 0.012 | 0.024 | -0.009 | -0.001 |
| Pregnancy depression score | 0.095 | -0.118 | 0.010 | -0.004 |
| Pregnancy computer use | -0.405 | 0.268 | -0.167 | 0.017 |
| Pregnancy phone use | -0.029 | 0.067 | -0.413 | 0.039 |
| Maternal age | -0.011 | 0.302 | 0.002 | -0.001 |
| Maternal race | -0.065 | -0.023 | -0.177 | 0.007 |
| Maternal educational level | 0.147 | -0.323 | 0.027 | 0.002 |
| Maternal occupation | -0.029 | 0.067 | 0 | 0.001 |
| Paternal age | -0.249 | -0.292 | -0.014**^*^** | 0.002**^*^** |
| Paternal race | -0.141 | -0.083 | 0.254**^*^** | 0.011 |
| Paternal educational level | -0.081 | -0.352 | -0.086 | 0.020**^*^** |
| Paternal occupation | -0.286 | -0.118 | -0.026 | 0.004 |

^*^Statistical significance
